# Mining morphometrics and age from past survey photographs

**DOI:** 10.1186/s12983-019-0309-x

**Published:** 2019-05-13

**Authors:** Caitlin E. Black, Hannah S. Mumby, Michelle D. Henley

**Affiliations:** 1 Bull Elephant Network Project, UCCRI, Department of Zoology, David Attenborough Building, Cambridge, UK; 2 Wissenschaftskolleg zu Berlin, Berlin, Germany; 3 Centre for African Ecology, University of Witwatersrand, Johannesburg, South Africa; 4 Applied Behavioural Ecology and Ecosystem Research Unit, Florida Campus, University of South Africa, Johannesburg, South Africa; 5 Elephants Alive, Hoedspruit, South Africa; 6 Present address: Department of Evolutionary Biology and Environmental Studies, University of Zurich, Zurich, Switzerland; 7 School of Biological Sciences, University of Hong Kong, Pok Fu Lam Road, Hong Kong SAR, China

**Keywords:** Body size, Images, Morphology, Hunting, African elephants, *Loxodonta africana*, Savanna elephant, Tusk, Conservation

## Abstract

**Background:**

Researchers often document wildlife surveys using images. These images contain data that can be used to understand alterative research objectives, even years after they were originally captured. We have developed a method to measure age and morphology (body size measurements and tusk size) from survey image databases and future surveys, without the availability of a known subject distance or a scale in each image. African savanna elephants (*Loxodonta africana*) serve as an ideal model species to develop a non-invasive, image-based morphometric methodology: as handling these animals is particularly invasive and expensive, involving anaesthesia and because of their IUCN ‘vulnerable’ status. We compare in situ measurements, taken during collaring events, to tusk-to-body-size ratios, measured from the images.

**Results:**

We provide evidence that relative morphological measurements, musth timing, and age of male African savanna elephants can accurately be obtained from a survey image database of over 30,000 images, taken over an 18-year period. Of the 11 tusk to body size ratios calculated, we recommend the use of two in particular for future measurement in African elephants to determine size and age: 1) tusk length to tusk diameter and 2) tusk length to body height.

**Conclusions:**

We present a practical, non-invasive measure to estimate morphometrics, including both age and tusk size from photographs, which has conservation applications to the protection of elephants and is relevant to a range of other taxa.

**Electronic supplementary material:**

The online version of this article (10.1186/s12983-019-0309-x) contains supplementary material, which is available to authorized users.

## Background

Ad libitum surveying of wildlife serves as a standard and universal method to count populations, identify and track individuals, and record survival [43]. These surveys are often photographed to allow for later analyses, which results in databases of images filed away after serving their initial purpose [12]. However, these images may contain additional valuable information, which can solve secondary objectives, even years after the original survey effort. In particular, obtaining morphometric data is fundamental to the study of ecology and evolution, allowing researchers to understand taxonomy, evolutionary divergence, mate choice, growth and development, and individual condition [11]. Although morphological measurements from images are routine in the lab setting [26], few studies have attempted to extract morphometrics from photographs in situ in wild animals [27]. Given the abundance of photographs available from ecological studies, optimizing survey efforts by extracting morphometric data could provide researchers with tangential information important to their study species and system.

Past studies have attempted to retrieve wildlife morphological information from images or still video by either 1) placing a scale in each image (e.g. [41, 42]), 2) photographing the subject at a known, fixed distance ([6, 20, 37]), 3) comparing the subject’s target morphology to known morphometrics (e.g. length of fish prey compared to bill length, [21]) or 4) by undergoing geometric morphometric analysis, which uses easily identifiable landmark points on the subject’s anatomy to create a 3-dimensional coordinate system to measure volume of morphology (e.g. beak shape in seven species of Darwin’s finches; [13]). Although these methods are practical to study specific species in situ, each research group either had access to reference measurement, obtained by handling the animals, or photographed the subjects at a fixed, known distance; this is not feasible when attempting to understand morphometrics from identification survey images because these measures are not typically recorded. Mahendiran et al. [27] designed a methodology to measure morphology in situ by extracting the distance from the lens to the subject from each image’s metadata. Although this method provides researchers with a framework to obtain morphometrics from survey images in future studies, the compulsory metadata is often not available from old images (e.g. in our study, only 6 of 32,296 images had this metadata recorded), as the subject-distance is only recorded in specific cameras and lens systems (e.g. Canon EOS 5D Mark III; [27]). Therefore, to measure morphology from survey image databases - without the availability of the subject distance or a scale in each image - we must develop and validate an alternative methodology.

African savanna elephants (*Loxodonta africana*) serve as an ideal species to develop a non-invasive, image-based morphometric methodology: as handling these animals is particularly invasive and expensive, involving anaesthetics [44]. In addition, African savanna elephant’s IUCN ‘vulnerable’ status [4] and unsustainable overall decline of 8% each year [7] makes the African elephant a conservation priority. Large-tusked males are particularly vulnerable to ivory poaching and trophy hunting due to their attractive large size [3], yet the length or weight of most tusks and the individual’s age is primarily determined observationally and therefore subjective (Henley pers. comm. [18]). These large males are integral to the health of the population; they contribute disproportionately more to the population’s genetic and social structures [1, 8, 35]. Where hunting is legal, protocols mandate hunted males be of certain age (e.g. 20 to 35 years in the Associated Private Nature Reserves; [38]) to ensure the protection of males in their reproductive prime (35 to 55 years; [38]). Hunting quotas and the cost of trophy hunting large-tusked males, which provide funds for the conservation of both elephants and other species, are often dependent on the size of the tusks [28]; therefore, knowledge of an elephant individual’s tusk dimensions prior to licensed hunts could provide evidence for larger fees and identify unsustainable practices. Objectively identifying individuals by size and age is essential information for ecologists, reserve managers, conservation institutions, and hunters themselves, to understand the age structure of a population, which males to prioritize for protection, and to ensure that males are accurately aged if they are to be hunted.

Data on fitness-relevant physiological states can also potentially be extracted from photographs. For example, musth is a period of elevated androgens production (male sex hormones) associated with sexual activity and aggressive behaviour [31]. In older elephants, a yearly musth cycle often forms, when males roam large distances away from their home ranges and are most attractive to females [30]. The timing of this period is linked to individual fitness and condition and determines when the individual may be more exposed to human-wildlife conflict [17]. Musth timing is determined by behavioural and visual cues in the field, including aggressive advances, urine dribbling, temporal gland swelling, and temporal gland secretions, which can be broken up into pre-, peak, and post-musth phases [16, 17]. However, studies have not yet focused on using visual cues to examine musth timing from photographs, which would provide researchers with a tool to understand individual condition and likelihood of mating success, as musth males outrank larger, more dominant males who are not in musth when competing for females [17].

Here, we test whether morphometric measurements obtained from a long-term photographic dataset of elephant can provide accurate age and relative tusk size indices by comparing the results with in situ measurements. Once established, we determine whether this method is free of random errors and how the results are influenced by the visibility of the focus individual’s body profile, photo quality, camera type and photographic settings. Lastly, we determine whether images can provide valuable information on the reproductive timing of males, using the criteria set by biologists to assess musth in the African elephant.

## Results

According to the criteria set, 2013 total images of 406 individuals were annotated out of a total 32,296 survey photos of 867 known-aged individuals taken between 2003 and 2017. Not all individuals photographed were also measured in situ; 22 individuals were either measured or aged in situ out of a total of 406 individuals that were measurable from survey photographs.

### Effects of visible body parts and blurry images

Welch two sample t-tests revealed significant differences between images where main anatomy was not visible compared to when all anatomy was visible in an image (or only the foot or anal flap were not visible) for all ratios that included body length (Additional file 1: Table S1). As a result, images where main anatomy was not visible were not included in future analyses (*n* = 205), except for our analysis of blurry vs. focused images.

Without removing annotations from images where main anatomy was not visible, Welch two sample t-tests revealed that blurry images were significantly different from images in focus for one of the 7 ratio variables: tusk diameter to body length (t = − 2.88, d.f. = 166, *p* < 0.004; Additional file 2: Table S2). As a result, all blurry images were removed from further analyses (*n* = 391). The final data subset resulted in 1417 images of 362 individuals, representing 4.39% of the original images (*n* = 32,396) and 41.75% of individuals (*n* = 867) in the survey database. This subset of images was used to understand intra-rater reliability, the effects of camera parameters, and body size estimation. However, a smaller subset of this data was used to 1) determine the validity of image ratios in comparison to in situ measurements and 2) age estimations, due to a limited number of individuals with in situ measurements available (*n* = 12–15 and *n* = 11, respectively).

### Intra-rater reliability

All ratios measured indicated excellent intra-class correlation (ICC) values (ICC > 0.75, *p* < 0.001) and therefore credible intra-rater reliability with the exception of the tusk diameter to foot diameter ratio (good ICC value, 0.60 < ICC < 0.74, *p* < 0.001; [9]; Additional file 3: Table S3).

### Validity of image ratios in comparison to in situ measurements

**Fig. 1:**
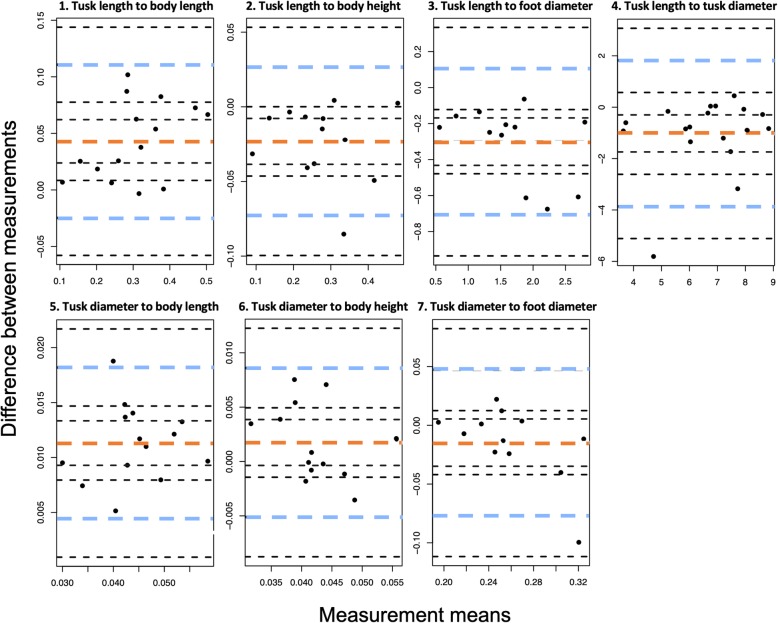
Bland-Altman plots for each of 7 ratios measured, comparing the means and differences of morphometrics obtained from survey images to ‘gold standard’ measurements obtained in situ from anesthetised elephants during collaring events. The ratios include: **1**) tusk length to body length, **2**) tusk length to body height, **3**) tusk length to foot diameter, **4**) tusk length to tusk diameter, **5**) tusk diameter to body length, **6**) tusk diameter to body height, and **7**) tusk diameter to foot diameter. The thick dashed orange lines represent mean differences and the thick blue dashed lines represent the upper and lower limits of agreement (LOA)

Bland-Altman tests revealed that all 7 ratios measured fall within the limits of agreement with a percent error of < 5%, with the exception of tusk diameter to foot diameter (lower limit percent error = 5.13%, Fig. 1, Table 1). Therefore, the ratios calculated from morphology measured in images- with the exception of tusk diameter to foot diameter- did not vary significantly from ‘gold standard’ morphology ratios obtained from tranquilized elephants during collaring events and are considered valid.

**Table 1:** Statistical results of Bland-Altman plots for each of 7 ratios measured, comparing the means and differences of morphometrics obtained from survey images to ‘gold standard’ measurements obtained in situ from darted elephants during collaring events. The lower and upper limit values indicate the limits of agreement (LOA). The percent error is calculated by dividing the limits of agreement by the mean value of the measurements. *n* = 12–15

Variable (ratio)	Mean differences	Lower limit			Upper limit		
			Percent error	95% CI		Percent error	95% CI
Tusk length: body length	0.043	−0.025	− 0.58	− 0.058 – 0.008	0.111	2.58	0.078–0.144
Tusk length: body height	− 0.023	− 0.073	3.17	− 0.100 – − 0.046	0.027	−1.17	5.74 × 10^− 5^ – 5.33 × 10^− 2^
Tusk length: foot diameter	−0.301	− 0.707	2.35	− 0.936 – − 0.479	0.105	−0.349	− 0.122 – 0.335
Tusk length: tusk diameter	−1.020	−3.86	3.78	−5.11 – − 2.61	1.823	− 1.787	0.574–3.07
Tusk diameter: body length	0.011	0.004	0.364	9.31 × 10^− 4^ – 0.080	0.018	1.636	0.015–0.022
Tusk diameter: body height	0.002	−0.005	− 2.50	− 0.009 – − 0.001	0.009	4.5	0.005–0.012
Tusk diameter: foot diameter	− 0.015	−0.077	5.13	−0.112 – − 0.042	0.047	− 3.13	0.012–0.082

A Cohen’s kappa test comparing musth observations from field surveys in situ to observations from survey images revealed a substantial agreement (κ = 0.73). The test also determined a sensitivity (proportion of musth males correctly identified by the test) of 0.972, a specificity (proportion of non-musth males correctly identified by the test) of 0.713, a positive predictive value (PPV; proportion of musth males in musth in situ) of 0.918, and a negative predictive value (NPV; proportion of non-musth males not in musth in situ) of 0.885.

### Body size estimation

Our two mixed-effects models, examining relationships between head measurements (head height and head girth in pixels) and the three body size measurements (shoulder height, body length, and foot diameter, also in pixels) showed positive significant relationships between head girth and foot diameter in all age classes (*p* = < 0.001) and head height and foot diameter in individuals over 25 years old (adults, prime adults, and senescing adults; Additional file 5: Table S5). The models did not reveal significant relationships between head height or girth and shoulder height or body length (Additional file 5: Table S5).

### Age estimation

**Fig. 2:**
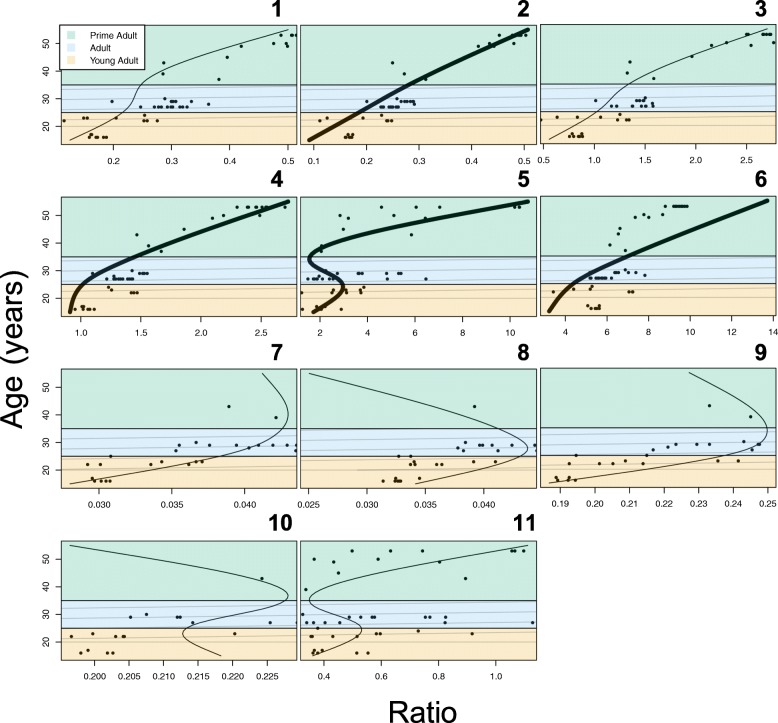
Calibration graphs of 11 linear mixed-effects models, showing the relationship between age, calculated from molar exams, and each of the 11 tusk to body size ratios, calculated from images, including a natural cubic spline (d.f. = 3) and individuals as random effects: (**1**) tusk length to body length, **2**) tusk length to body height, **3**) tusk length to foot diameter, **4**) tusk length to head height, **5**) tusk length to head girth, **6**) tusk length to tusk diameter, **7**) tusk diameter to body length, **8**) tusk diameter to body height, **9**) tusk diameter to foot diameter, **10**) tusk diameter to head height, and **11**) tusk diameter to head girth. Thick lines represent models with significant *p* values < 0.05. Each age class represented by different coloured backgrounds: **1**) young adults from 15 to 25 years in orange, **1**) adults from 25 to 35 years in blue, and **3**) prime adults from 35 to 55 years in green. The current recommended age for sustainably hunting males based on local protocol depicted by grey dashed line. *n* = 11 individuals

Our 11 linear mixed-effects models, examining the relationships between photo ratios and age, as determined from molar examinations, revealed 4 ratios in which all three cubic spline values were statistically significant (*p* < 0.05; Table 2). In other words, in these 4 ratios, the predicted values - resulting from a smoothing best-fit model - did not differ significantly from the actual values, calculated from the photographs and molar examinations, in the entire cubic spline curve. These ratios are 1) tusk length to body height, 2) tusk length to head height, 3) tusk length to head girth, and 4) tusk length to tusk diameter (Table 2, Fig. 2).

**Table 2:** Results from 11 linear mixed-effects models explaining the relationship between age, calculated from molar exams, and each of the 11 tusk to body size ratios, calculated from images, with a natural cubic spline (d.f. = 3) and individuals as random effects. *represents explanatory variables with significant p values (< 0.05) for all three cubic spline values. n = 11 individuals

Explanatory variable (ratio)	Cubic spline	Value	Standard error	DF	*p* value
Tusk length: body length	1	0.060	0.071	43	0.399
	2	0.330	0.169	43	0.058
	3	0.277	0.071	43	< 0.001
Tusk length: body height	1	0.165	0.046	40	0.001*
	2	0.377	0.118	40	0.003*
	3	0.340	0.047	40	< 0.001
Tusk length: foot diameter	1	0.530	0.308	40	0.093
	2	1.80	0.751	40	0.021
	3	1.71	0.308	40	< 0.001
Tusk length: head height	1	0.610	0.251	40	0.019*
	2	1.25	0.617	40	0.050*
	3	1.65	0.251	40	< 0.001
Tusk length: head girth	1	−2.96	1.86	40	0.120*
	2	4.75	2.13	40	0.031*
	3	6.70	0.979	40	< 0.001
Tusk length: tusk diameter	1	4.02	1.33	40	0.004*
	2	8.05	3.44	40	0.024*
	3	9.31	1.35	40	< 0.001
Tusk diameter: body length	1	0.011	0.006	40	0.098
	2	0.023	0.013	40	0.080
	3	0.007	0.006	40	0.204
Tusk diameter: body height	1	0.002	0.006	40	0.731
	2	0.007	0.015	40	0.662
	3	−0.013	0.006	40	0.033
Tusk diameter: foot diameter	1	0.041	0.032	40	0.202
	2	0.094	0.069	40	0.181
	3	0.012	0.030	40	0.690
Tusk diameter: head height	1	0.026	0.025	40	0.315
	2	−0.007	0.061	40	0.911
	3	−0.012	0.025	40	0.646
Tusk diameter: head girth	1	−0.334	0.225	40	0.146
	2	0.442	0.236	40	0.068
	3	0.500	0.106	40	< 0.001

### Effects of camera parameters

An interaction between aperture and shutter speed showed a significant positive relationship with the accuracy of the tusk diameter to body height (*p* = 0.011) and tusk diameter to body height (*p* = 0.039) ratios. In other words, a large aperture and fast shutter speed served as the ideal settings for measuring two of the tusk to body size ratios measured. White balance during daylight also significantly influenced the accuracy of the tusk length to body length ratio (*p* = 1.510 × 10^− 8^) and tusk length to tusk diameter (*p* = 0.040).

The camera models Canon IXUS 330 (*p* = 0.033), Canon PowerShot A95 (*p* = 0.034), and Nikon D70 (*p* = 0.014) were significantly more accurate than the other camera models at measuring the tusk length to body length and tusk diameter to body height ratios (Additional file 4: Table S4).

## Conclusions

We provide evidence that age and relative morphological measurements can accurately be obtained from a survey image database and from future survey efforts. Our results indicate that this non-invasive method for measuring relative morphology, age, and musth timing is valid with great accuracy, when compared to in situ measurements. Moreover, the database, originally created to identify individuals, provided morphometric information from 41.75% of individuals. Although this method does not provide information on traditional morphometric measurements (e.g. tusk length in cm), the method does allow for information on relative tusk size to compare individuals. This methodology has potential to provide ecologists, reserve managers, and hunters with a tool to objectively identify the age and relative size of large, prime-aged males to best conserve these individuals who are most likely to contribute to the population’s genetic and social structure [1, 8, 35].

Of the 11 tusk and body size ratios calculated, we recommend the use of two ratios in particular to measure African elephants in future studies: tusk length to tusk diameter and tusk length to body height. The tusk length to body height ratio proved to be valid compared to ratios calculated from in situ field measurements (− 1.17–3.17% error; Table 1) and in our aging model (*p* ≤ 0.003; Table 2), so can accurately be used to compare tusk size between individual elephants in future studies. Likewise, the tusk length to tusk diameter ratio was also a valid method to age elephants with all three cubic spline values being statistically significant (*p* < 0.024; Table 2, Fig. 2) and valid compared to measurements taken during collaring events (− 1.787–3.78% error, Table 1). This ratio has been used to age elephants in the past [32] and we also recommend that this ratio be calculated when aging elephants and comparing tusk sizes across individuals. Future studies could use a larger sample size of individuals directly measured and aged at the same time in order to confirm this approach.

In elephants, survey photographs often only focus on an individual’s head because researchers aim to document the ear pattern for individual recognition. Therefore, measuring tusks - rather than body sizes - may allow this method to be applied to a larger sample size, including additional study sites where photographs of the full body in profile are an anomaly. It is also important to note that future studies should discard blurry images from data sets and that camera models and settings do not affect the accuracy of ratio measurements (Additional file 4: Table S4). These findings further validate the method’s relevance for use in other survey databases and collaborative studies in which camera settings, as is the case in our study, are particularly diverse and rarely standardized across a long-term study period or multiple field sites.

Given the numerous uses of morphometrics in ecological studies and the various types of photographic surveys, this method has high potential to extract secondary data from a suite of survey databases. As past studies have used morphometrics to estimate body weight, to understand the adaptive significance of ornaments, to recognize variation within communities, and to identify adaptive radiation in a range of taxa, understanding ecomorphology at the individual, population, and species levels provides insight into both the proximate and ultimate cause of behaviours [24]. In addition, morphometrics can be used to estimate body mass [10], which is particularly relevant in African elephants to understand the size of an individual before tranquilizing it for relocation purposes; however, a formula to estimate weight from morphometrics does not yet exist and should be a priority for future research. Currently, a consensus for the tusk size requirements of large ‘tuskers’ does not exist throughout the species range, even though large-tusked males are essential members of social networks and contribute disproportionately to population genetics [1, 8, 35]. Future studies should aim to determine minimum tusk measurements and weights for ‘tuskers,’ which could then provide a baseline tusk size ratio to determine ‘tuskers’ from images. By obtaining data on tusk morphometrics, in particular, images have the potential to provide detailed insight into the relationship between tusk size and sociality, dominance, condition, hormones and phenology, all of which are associated with individual fitness in this species [31]. In addition to survey photographs, morphometrics can likely be obtained from a range of additional images, including aerial photographs, satellite images, camera traps, and even tourist photographs. This novel methodology is particularly relevant when examining species with different rates of decline, spanning large native ranges, and with diverse management strategies, as is the case with the African savanna elephant.

## Methods

### Study site

**Fig. 3:**
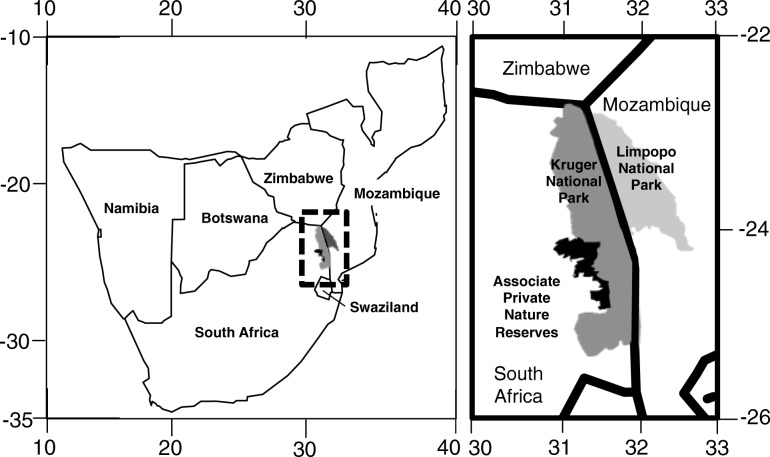
Location of the Associated Private Nature Reserves (APNR) study site in South Africa

The study was conducted in the Associated Private Nature Reserves (APNR) in South Africa, an ~ 1800 km^2^ area unfenced to Kruger National Park, which includes Balule (24°9′0″ S, 30°59′0″ E), Klaserie (24°15′23″ S, 31°13′1″ E), Timbavati (24°20′7″ S, 31°20′38″ E), and Umbabat (24°9′8″ S, 31°22′16″ E) private nature reserves (Fig. 3). A 2017 census estimated a population of 2224 elephants (male and female) in the APNR [22].

### Photographic surveys

Photographic surveys were officially conducted from 2003 to 2017 ad libitum 2–3 days each week (mean = 2.88 between 2003 and 2013) by vehicle by the nongovernmental organization *Elephants Alive* within the APNR. The initial purpose of the survey photographs was to identify individuals based on ear patterns; therefore, many of the images contained a small frame of view centred on individual ears. A total of 54 different camera models were used over the study period with a variety of camera settings in JPEG format (Additional file 4: Table S4).

### In situ aging and size measurements

**Fig. 4:**
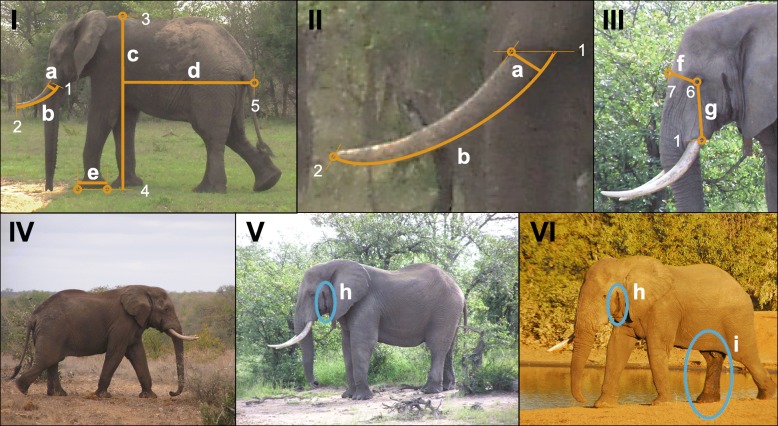
Tusk and body size measurements and musth indications possible in each image. Tusk and body size measurements obtainable in each image include (**I**-**II**) a) tusk diameter at lip line (1), b) tusk length from lip line to tip (2), c) shoulder height from scapula (3) to bottom of straight forelimb in foreground (4), d) body length: horizontal length from scapula (3) to anal flap (5), and e) foot diameter during stride). Facial features measurable in pixels in each image include (**III**) f) head girth measured from eye tear gland (6) to nasal cavity (7) and g) head height measured from tear gland to lip line. Indicators of non-musth (**IV**) or musth period (V-VI) distinguishable in images include temporal gland secretions (h) and urine dribbling (i)

Tusk and body size measurements (tusk length, tusk circumference, body length, shoulder height, and foot length; Fig. 4) were obtained in situ while individuals were sedated during GPS collaring events over the 18-year study period, as described in Ngure [29].

From 2005 to 2010, 11 males were each aged by three separate individuals, based on standard molar evaluation protocol, during unique collaring events [34]. For each image, the individual’s age was then determined, using the year of the collaring event when aging took place as a baseline (e.g. an individual aged as 25 in 2005 was noted as 30 in a 2010 photograph). In situ tusk circumference measurements were converted to tusk diameter by dividing the measurement by π. Not all individuals photographed were also measured in situ; 22 individuals were either measured or aged in situ out of a total of 406 individuals that were measurable from survey photographs.

### Image annotations

Individuals were aged and identified by ear patterns, first in the field and later confirmed from the photographs, using the methods described in Poole [31]. We categorized each individual by age class, using physical and behavioural characteristics, including sub-adults (< 15 years), young adults (15–25 years), adults (25–35 years), prime adults (35–55 years), or senescing adults (> 55 years; [19]). Images within this database were organized to include only images of male elephants where tusks (Fig. 4a, b) and at least one body dimension was in view (Fig. 4c, d, e).

Using Adobe Illustrator CC (v21.1.0; Adobe Systems Inc., San Jose, CA, USA), the following were measured in pixels, (a-e defined in Lindeque and Van Jaarsveld [25], Poole [31], and Fowler and Mikota [14]):

- Tusk diameter: length of tusk at lip line (Fig. 4a).
- Tusk length: length of tusk from lip line (1) to tip (2) along rounded edge (Fig. 4b).
- Body height (aka. shoulder height): vertical length from scapula (3) to bottom of straight forelimb in foreground (4, Fig. 4c).
- Body length (aka. back length): horizontal length from scapula (3) to anal flap (5, Fig. 4d).
- Foot Diameter: length of bottom of straight forelimb in foreground (Fig. 4e).
- Head girth: length from the tear gland (6) to nasal cavity (7, Fig. 4g).
- Head height: length from tear gland (6) to lip line (1, Fig. 4h)

For each image, we also noted the following visible musth signs (Fig. 4h) temporal gland secretion (TGS) and i) urine dribbling (UD; Fig 4i [16]). Temporal swelling was not recorded, although it is an indicator of musth [16], because swelling was difficult to objectively identify in photographs taken from varying angles and in different lighting conditions. It is important to note that elephants can exhibit temporal gland secretions when they are excited or stressed, not only when they are in musth [5].

Lastly, the quality of the image was noted for the following categories:

- Anatomy not visible: if any of the following were not visible in an image, it was noted (Fig. 4): 1) lip line, 2) tusk tip, 3) scapula, 4) foot bottom, and 5) anal flap.
- Blurred Images: if the image was out of focus, blurry, low resolution, or the individual was too small in the frame to measure anatomy, it was noted.

### Image metadata

The following metadata were obtained from all unaltered images using ExifTool software (Harvey 2013): 1) JPEG (nominal), 2) date, 3) time 4) aperture (f-stops; discrete), 5) camera model (nominal), 6) focal length (mm; discrete), 7) ISO (discrete), 8) megapixels (discrete), 9) quality (nominal), 10) shutter speed (seconds; continuous), and 11) white balance (nominal).

### Statistical analysis

For each individual, the following ratios were computed from measurements in pixels:

1. Tusk length: body length
2. Tusk length: body height
3. Tusk length: foot diameter
4. Tusk length: head height
5. Tusk length: head girth
6. Tusk length: tusk diameter
7. Tusk diameter: body length
8. Tusk diameter: body height
9. Tusk diameter: foot diameter
10. Tusk diameter: head height
11. Tusk diameter: head girth

Before conducting statistical analyses, outliers for each ratio were determined and re-measured. All statistics were conducted using R [36].

#### Effects of blurry images and visible body parts

Welch two sample t-tests (*t.test* function, *stats* package) were conducted for each of 9 ratios (not including head girth and head height ratios) to determine whether a difference existed between images in focus and blurry images. The t-tests were repeated for each ratio to determine whether a difference existed between images with either 1) body length, 2) body length, or 3) foot diameter visible and those where either the 1) scapula 2) anal flap and foot, 3) scapula and anal flap, or 4) scapula, foot, and anal flap were not visible. A Bonferroni correction (*p* value of 0.05/number of t-tests) indicated that a *p* value of < 0.005 represents significance.

#### Intra-rater reliability

Morphometrics were measured twice by the same observer in 10% of the images (*n* = 202) to determine the intra-rater reliability (IRR, aka. within-observer reliability or observer consistency). IRR was assessed with two-way, absolute agreement, single-measures intra-class correlation (ICC) models for each of the 11 ratios [40], using the *icc* function in the *irr* package [15].

#### Validity of image ratios in comparison to in situ measurements

To determine the validity of each of the 7 ratios (not including head girth and head height ratios as these were not measured during collaring events), Bland-Altman tests were conducted comparing 1) ratios calculated for each individual obtained from one image to 2) ratios of the same individuals obtained in situ during collaring events (*n* = 12–15; *bland.altman.stats* function in *BlandAltmanLeh* package; [23, 40]). The image which was taken closest in time to the collaring event (mean = 13.8 days) and that allowed us to calculate the most ratios (based on the angle of the individual and the body parts visible) was used in our validity calculations. We determined a priori criteria of 5% error to measure the validity. The percentage error was subsequently obtained by dividing the limits of agreement (lower and upper limits) by the mean differences value [40].

To understand whether musth signs can be determined from images, we compared sightings where musth/non-musth was recorded in situ to photographs from the same day. The musth status was determined in the photograph before the in situ musth status of the individual was revealed to the measurer. Any in situ observations marked as “not sure” were removed from our analysis and males with either TGS or TGS/UD observed in images were deemed in musth. A Cohen’s kappa test was then conducted on the contingency table with musth as a binary number, using the *cohen.kappa* function in the *psych* package [2, 40].

#### Body size estimation

To understand whether head height or head girth (measured in pixels, Fig. 4) could be used to estimate body size, we ran a linear mixed-effects models for each head measurement as a function of each of the three body measurements without interaction terms (body height, body length, and foot diameter; *lme* function in the *nlme* package; [33]; Fig. 4). Because of repeated measures, individuals were entered into the models as a random effect and nested within age class. After visually inspecting quantile-quantile plots (*qqnorm* function in *stats* package), we included all age classes in the head girth model but included only individuals over 25 years old (adults, prime adults, and senescing adults) in the head height model due to a large skew in the standardized residuals in younger individuals.

#### Age estimation

To determine whether age, as determined from molar examinations, can accurately be predicted by calculating ratios from survey images, we used the *lme* function (Bates et al. 2012) in the *nlme* package [33] to perform linear mixed-effects analyses. Each of 11 models included the photo ratio as a response variable and the known age on the date the photo was taken as an explanatory variable. A natural cubic spline (d.f. = 3) was also included in each model to fit a smooth curve to the data for visualization purposes (*ns* function in *splines* package; [39]). Because of repeated measures, individuals were entered into the models as a random effect. Visual inspection of quantile-quantile plots (*qqnorm* function in *stats* package) did not reveal any obvious deviations from normality. Calibration curves were then plotted using the predicted values resulting from each model (*predict* function, *stats* package) to form a best-fit line over the actual data points (Fig. 2). The x and y axis were reversed in the calibration graphs (Fig. 2) for graphical purposes only to show how photograph ratios can be used to determine age in future studies by following the best-fit line.

#### Effects of camera parameters

To determine whether camera parameters, obtained from image metadata, influence the accuracy of ratio measurements, we subtracted mean differences derived from Bland-Altman tests from each of the 7 ratios (not including head girth and head height ratios; Table 1). We then ran the following generalized linear models (GLMs) with Gaussian distributions for each of the 7 ratios with these new accuracy scores (deviation from the mean difference) as response variables (*glm* function in *stats* package).

$$ Accuracy\ score\sim Megapixels+{Aperture}^{\ast }\ {Shutter\ speed}^{\ast }\  ISO+ White\ balance+ Focal\ length+ Quality+ Camera\ model $$

*P*-values underwent Bonferroni corrections using the *p.adjust* function in the *stats* package to avoid type I errors.

## Additional files

Additional file 1: **Table S1.** Welch two sample t-tests compared survey images where main anatomy was not visible (anal flap, foot; scapula; scapula, anal flap; scapula, anal flap, foot;scapula, foot) vs. when either 1) body length, 2) body height, or 3) foot diameter was clearly visible in an image for each of 6 relevant morphometric variables (represented by ratios). A Bonferroni correction indicated that a *p* value of < 0.005 represents significance. (PDF 17 kb)

Additional file 2: **Table S2.** Welch two sample t-tests compared each of 7 morphometric variables (represented by ratios) measured from survey photographs with and without the inclusion of blurry images. A Bonferroni correction indicated that a *p* value of < 0.005 represents significance. (PDF 17 kb)

Additional file 3: **Table S3.** Two-way, absolute agreement, single-measures intra-class correlation (ICC) models assessed intra-rater reliability for each of 11 morphology measurements obtained from survey photographs. *p* < 0.001 for all ICC models. (PDF 21 kb)

Additional file 4: **Table S4.** Results from two linear mixed-effects models with 1) head girth and 2) head height as response variables and body size (body height, body length, and foot diameter) as explanatory variables, with individuals nested within age categories included as random effects. The head girth model includes all age categories while the head height model only includes individuals over 25 years old (adults, prime adults, and senescing adults). All variables measured in pixels. * indicates significant *p* values < 0.05. (PDF 115 kb)

Additional file 5: **Table S5.** Output of generalized linear models for each of 7 explanatory variables, represented as ratios. * represents significant Bonferroni adjusted *p* values. Positive estimates indicate a positive relationship between coefficient and errors (ratio - mean difference derived from Bland-Altman test). (PDF 18 kb)

Additional file 6: Collaring procedures. (DOCX 25 kb)
