# Supplementary material for: Mining morphometrics and age from past survey photographs
Source: Front Zool. 2019 May 13;16:14. doi: 10.1186/s12983-019-0309-x (PMC6513526; doi:10.1186/s12983-019-0309-x)
Supplement: Supplementary file 1 — Table S1. Welch two sample t-tests compared survey images where main anatomy was not visible (anal flap, foot; scapula; scapula, anal flap; scapula, anal flap, foot;scapula, foot) vs. when either 1) body length, 2) body height, or 3) foot diameter was clearly visible in an image for each of 6 relevant morphometric variables (represented by ratios). A Bonferroni correction indicated that a p value of < 0.005 represents significance. (PDF 17 kb) [file 12983_2019_309_MOESM1_ESM.pdf]

Supplementary Table 1.

Variable (ratio)	t	d.f.	*p* value	95% CI	$Mean_{Visible}$	$Mean_{Not\ Visible}$
Tusk length: body length	-3.43	208	<0.001	-0.072 – -0.0195	0.304	0.349
Tusk length: body height	0.238	0.038	0.271	-0.011 – 0.0385	0.238	0.224
Tusk length: foot diameter	0.720	76.1	0.474	-0.091 – 0.1945	1.42	1.37
Tusk diameter: body length	-6.03	212	<0.001	-0.008 – -0.0040	0.049	0.55
Tusk diameter: body height	0.048	80.6	0.962	-0.003 – 0.0029	0.040	0.040
Tusk diameter: foot diameter	-0.471	73.8	0.639	-0.018 – 0.0113	0.236	0.239
