# Supplementary material for: Mining morphometrics and age from past survey photographs
Source: Front Zool. 2019 May 13;16:14. doi: 10.1186/s12983-019-0309-x (PMC6513526; doi:10.1186/s12983-019-0309-x)
Supplement: Supplementary file 2 — Table S2. Welch two sample t-tests compared each of 7 morphometric variables (represented by ratios) measured from survey photographs with and without the inclusion of blurry images. A Bonferroni correction indicated that a p value of < 0.005 represents significance. (PDF 17 kb) [file 12983_2019_309_MOESM2_ESM.pdf]

Supplementary Table 2.

Variable (ratio)	t	d.f.	p-value	95% CI	$Mean_{Not\ Blurry}$	$Mean_{Blurry}$
Tusk length: body length	-1.43	179	0.154	-0.031 – 0.005	0.304	0.317
Tusk length: body height	-0.389	64.3	0.699	-0.036 – 0.024	0.236	0.242
Tusk length: foot diameter	-0.136	62.4	0.892	-0.195 – 0.170	1.41	1.43
Tusk length: tusk diameter	1.20	215	0.230	-0.985 – 0.407	6.14	5.98
Tusk diameter: body length	-2.88	166	0.004	-0.005 – -8.93×10^{-4}	0.049	0.052
Tusk diameter: body height	-1.02	63.2	0.314	-0.005 – 0.002	0.039	0.041
Tusk diameter: foot diameter	-0.485	61.4	0.630	-0.023 – 0.014	0.236	0.241
