# Supplementary material for: Mining morphometrics and age from past survey photographs
Source: Front Zool. 2019 May 13;16:14. doi: 10.1186/s12983-019-0309-x (PMC6513526; doi:10.1186/s12983-019-0309-x)
Supplement: Supplementary file 3 — Table S3. Two-way, absolute agreement, single-measures intra-class correlation (ICC) models assessed intra-rater reliability for each of 11 morphology measurements obtained from survey photographs. p < 0.001 for all ICC models. (PDF 21 kb) [file 12983_2019_309_MOESM3_ESM.pdf]

Supplementary Table 3.

Ratio measured	ICC	Subjects	95% CIs
Tusk length: body length	0.874	176	0.833 – 0.905
Tusk length: body height	0.990	173	0.987 – 0.993
Tusk length: foot diameter	0.914	174	0.885 – 0.935
Tusk length: head height	0.998	139	0.997 – 0.998
Tusk length: head girth	0.980	129	0.971 – 0.986
Tusk length: tusk diameter	0.823	195	0.771 – 0.864
Tusk diameter: body length	0.929	178	0.896 – 0.950
Tusk diameter: body height	0.928	175	0.903 – 0.947
Tusk diameter: foot diameter	0.724	175	0.645 – 0.787
Tusk diameter: head height	0.992	139	0.989 – 0.994
Tusk diameter: head girth	0.975	129	0.965 – 0.982
