# Supplementary material for: Mining morphometrics and age from past survey photographs
Source: Front Zool. 2019 May 13;16:14. doi: 10.1186/s12983-019-0309-x (PMC6513526; doi:10.1186/s12983-019-0309-x)
Supplement: Supplementary file 4 — Table S4. Results from two linear mixed-effects models with 1) head girth and 2) head height as response variables and body size (body height, body length, and foot diameter) as explanatory variables, with individuals nested within age categories included as random effects. The head girth model includes all age categories while the head height model only includes individuals over 25 years old (adults, prime adults, and senescing adults). All variables measured in pixels. * indicates significant p values < 0.05. (PDF 115 kb) [file 12983_2019_309_MOESM4_ESM.pdf]

Supplementary Table 4.

Response variable	Coefficient	Estimate	Standard Error	*p* value	Adjusted *p* value
Tusk length: body length	Model Olympus C-740 UZ (Intercept)	0.309	0.021	2.000E-16	2.240E-45*
	Megapixels	-0.003	0.005	0.474	1.000
	Aperture	-0.001	0.003	0.595	1.000
	Shutter speed	-14.430	4.961	0.004	0.207
	ISO	-1.357E-5	2.512E-5	0.589	1.000
	White balance cloudy	-0.003	0.018	0.884	1.000
	White balance daylight	0.140	0.022	2.700E-10	1.510E-8*
	White balance manual	-0.055	0.035	0.119	1.000
	White balance shade	-0.057	0.018	0.002	0.107
	White balance sunny	-0.093	0.050	0.064	1.000
	White balance tungsten	-0.078	0.081	0.336	1.000
	Focal length	-1.530E-4	5.698E-5	0.007	0.410
	Quality low	-0.068	0.050	0.177	1.000
	Quality normal	-0.026	0.017	0.130	1.000
	Quality super fine	-0.347	0.128	0.007	0.383
	Model Olympus C-750 UZ	-0.023	0.010	0.019	1.000
	Model Olympus C-765 UZ	0.023	0.105	0.826	1.000
	Model Canon IXUS 330	-0.082	0.024	0.001	0.033 *
	Model Canon IXUS 55	-0.035	0.041	0.395	1.000
	Model Canon IXUS II	0.031	0.106	0.768	1.000
	Model Canon EOS 10D	0.161	0.117	0.168	1.000
	Model Canon EOS 1100D	0.034	0.055	0.534	1.000
	Model Canon EOS 300D	-0.024	0.080	0.764	1.000
	Model Canon EOS 350D	0.172	0.067	0.010	0.547
	Model Canon EOS 400D	-0.013	0.051	0.800	1.000
	Model Canon EOS 40D	0.218	0.113	0.054	1.000
	Model Canon EOS 650D	0.028	0.099	0.775	1.000
	Model Canon EOS 7D	-0.080	0.092	0.384	1.000

	Model Canon EOS REBEL XTi	0.172	0.064	0.007	0.399
	Model Canon PowerShot A95	-0.123	0.044	0.005	0.301
	Model Canon PowerShot S1 IS	0.160	0.106	0.133	1.000
	Model Canon PowerShot S2 IS	0.251	0.107	0.020	1.000
	Model Canon PowerShot S3 IS	0.289	0.132	0.028	1.000
	Model Canon PowerShot S40	0.310	0.140	0.027	1.000
	Model Canon PowerShot S410	0.501	0.167	0.003	0.150
	Model Canon PowerShot S45	0.090	0.077	0.241	1.000
	Model Canon PowerShot SX40 HS	0.013	0.070	0.849	1.000
	Model Sony DSC-HX 400V	0.074	0.116	0.522	1.000
	Model Olympus E-1	0.002	0.105	0.981	1.000
	Model Fujifilm FinePix S5000	-0.023	0.023	0.325	1.000
	Model Fujifilm FinePix S5600	0.053	0.018	0.004	0.237
	Model Fujifilm FinePix 4700	0.028	0.076	0.718	1.000
	Model Fujifilm FinePix S2 Pro	3.771E-4	0.052	0.994	1.000
	Model Kodak DX6490	0.059	0.040	0.141	1.000
	Model Nikon D100	-0.015	0.067	0.819	1.000
	Model Nikon D300S	0.004	0.065	0.955	1.000
	Model Nikon D3100	0.045	0.039	0.244	1.000
	Model Nikon D50	0.052	0.021	0.011	0.639
	Model Nikon D70	0.033	0.022	0.130	1.000
	Model Nikon D70S	-0.018	0.026	0.486	1.000
	Model Pentax *ist DL	0.207	0.079	0.009	0.497
	Model Casio QV-R41	-0.021	0.114	0.852	1.000
	Aperture: shutter speed	0.509	0.664	0.443	1.000
	Aperture: ISO	1.537E-7	1.781E-6	0.931	1.000
	Shutter speed: ISO	0.002	0.007	0.830	1.000
	Aperture: shutter speed: ISO	1.407E-5	0.001	0.980	1.000
Tusk length: body height	Model Olympus C-740 UZ (Intercept)	0.259	0.029	2.000E-16	1.54E-16*
	Megapixels	0.004	0.005	0.484	1.000
	Aperture	-0.008	0.004	0.053	1.000
	Shutter speed	-6.414	8.005	0.423	1.000
	ISO	-3.059E-4	2.074E-4	0.102	1.000

White balance cloudy	0.022	0.022	0.319	1.000
White balance daylight	-0.012	0.028	0.673	1.000
White balance manual	-0.029	0.039	0.463	1.000
White balance shade	0.021	0.026	0.426	1.000
White balance sunny	-0.038	0.056	0.503	1.000
Focal length	-1.503E-4	8.236E-5	0.069	1.000
Quality low	-0.046	0.040	0.259	1.000
Quality normal	-0.006	0.022	0.776	1.000
Quality super fine	0.133	0.067	0.049	1.000
Model Olympus C-750 UZ	-0.021	0.012	0.072	1.000
Model Canon IXUS 330	-0.047	0.035	0.172	1.000
Model Canon IXUS 55	0.010	0.047	0.834	1.000
Model Canon EOS 1100D	0.061	0.068	0.364	1.000
Model Canon EOS 300D	-0.030	0.052	0.566	1.000
Model Canon EOS 350D	0.060	0.069	0.386	1.000
Model Canon EOS 400D	0.035	0.066	0.597	1.000
Model Canon EOS 40D	0.129	0.081	0.110	1.000
Model Canon EOS 650D	-0.011	0.106	0.920	1.000
Model Canon EOS 7D	-0.008	0.112	0.945	1.000
Model Canon PowerShot A95	-0.092	0.045	0.040	1.000
Model Canon PowerShot S1 IS	0.212	0.090	0.019	0.827
Model Canon PowerShot S3 IS	-0.161	0.066	0.015	0.657
Model Canon PowerShot S40	-0.207	0.088	0.020	0.840
Model Canon PowerShot S410	NA	NA	NA	NA
Model Canon PowerShot SX40 HS	-0.071	0.107	0.510	1.000
Model Olympus E-1	0.004	0.091	0.963	1.000
Model Fujifilm FinePix S5000	-0.021	0.031	0.500	1.000
Model Fujifilm FinePix S5600	0.015	0.023	0.527	1.000
Model Fujifilm FinePix 4700	0.187	0.091	0.040	1.000
Model Fujifilm FinePix S2 Pro	-0.013	0.060	0.833	1.000
Model Nikon D300S	-0.029	0.079	0.718	1.000
Model Nikon D3100	0.069	0.048	0.150	1.000
Model Nikon D50	0.025	0.026	0.335	1.000

	Model Nikon D70	-0.013	0.027	0.630	1.000
	Model Nikon D70S	-0.005	0.042	0.897	1.000
	Aperture: shutter speed	-1.398	1.259	0.268	1.000
	Aperture: ISO	3.378E-6	2.193E-6	0.124	1.000
	Shutter speed: ISO	0.004	0.010	0.664	1.000
	Aperture: shutter speed: ISO	0.001	0.001	0.502	1.000
Tusk length: foot diameter	Model Olympus C-740 UZ (Intercept)	1.426	0.181	1.470E-13	6.33E-13*
	Megapixels	0.008	0.035	0.815	1.000
	Aperture	-0.031	0.026	0.228	1.000
	Shutter speed	-36.480	50.120	0.467	1.000
	ISO	-3.059E-4	2.074E-4	0.141	1.000
	White balance cloudy	0.181	0.141	0.199	1.000
	White balance daylight	-0.061	0.169	0.718	1.000
	White balance manual	-0.227	0.243	0.351	1.000
	White balance shade	0.012	0.161	0.941	1.000
	White balance sunny	-0.381	0.348	0.274	1.000
	Focal length	-0.001	0.001	0.011	0.478
	Quality low	-0.312	0.254	0.220	1.000
	Quality normal	-0.039	0.147	0.791	1.000
	Quality super fine	0.772	0.420	0.067	1.000
	Model Olympus C-750 UZ	-0.115	0.072	0.111	1.000
	Model Canon IXUS 330	-0.372	0.243	0.127	1.000
	Model Canon IXUS 55	0.072	0.282	0.799	1.000
	Model Canon EOS 1100D	0.339	0.442	0.443	1.000
	Model Canon EOS 300D	-0.179	0.398	0.653	1.000
	Model Canon EOS 350D	0.358	0.431	0.406	1.000
	Model Canon EOS 400D	0.442	0.636	0.488	1.000
	Model Canon EOS 40D	0.970	0.509	0.057	1.000
	Model Canon EOS 650D	0.081	0.680	0.906	1.000
	Model Canon EOS 7D	0.106	0.696	0.879	1.000
	Model Canon PowerShot A95	-0.524	0.335	0.118	1.000
	Model Canon PowerShot S1 IS	0.829	0.560	0.139	1.000
	Model Canon PowerShot S3 IS	-0.973	0.408	0.018	0.751

	Model Canon PowerShot S40	-1.156	0.677	0.088	1.000
	Model Canon PowerShot S410	NA	NA	NA	NA
	Model Sony DSC-HX 400V	0.292	0.869	0.737	1.000
	Model Olympus E-1	0.181	0.401	0.651	1.000
	Model Fujifilm FinePix S5000	-0.176	0.187	0.347	1.000
	Model Fujifilm FinePix S5600	0.058	0.150	0.701	1.000
	Model Fujifilm FinePix 4700	0.803	0.565	0.156	1.000
	Model Fujifilm FinePix S2 Pro	0.034	0.395	0.932	1.000
	Model Nikon D300S	-0.081	0.504	0.872	1.000
	Model Nikon D3100	0.456	0.307	0.138	1.000
	Model Nikon D50	0.275	0.163	0.092	1.000
	Model Nikon D70	-0.069	0.181	0.705	1.000
	Model Nikon D70S	-0.050	0.255	0.844	1.000
	Aperture: shutter speed	-7.637	7.733	0.324	1.000
	Aperture: ISO	1.678E-5	1.367E-5	0.220	1.000
	Shutter speed: ISO	0.039	0.062	0.533	1.000
	Aperture: shutter speed: ISO	0.002	0.005	0.700	1.000
Tusk length: tusk diameter	Model Olympus C-740 UZ (Intercept)	6.040	0.298	< 2E -16	< 0.001*
	ISO	-0.001	0.000	0.001	0.026*
	White Balance Cloudy	-0.459	0.292	0.116	1.000
	White Balance Daylight	1.229	0.367	0.001	0.040*
	White Balance Manual	-0.763	0.573	0.183	1.000
	White Balance Shade	-0.373	0.327	0.254	1.000
	White Balance Sunny	-0.333	0.828	0.688	1.000
	White Balance Tungsten	0.147	1.257	0.907	1.000
	Quality Low	-1.222	0.814	0.134	1.000
	Quality Normal	0.092	0.274	0.736	1.000
	Quality Super Fine	-5.173	2.468	0.036	1.000
	Model C750UZ	-0.154	0.164	0.347	1.000
	Model C765UZ	-0.566	2.019	0.779	1.000
	Model Canon DIGITAL IXUS 330	-0.639	0.403	0.113	1.000
	Model Canon DIGITAL IXUS 55	0.092	0.560	0.870	1.000
	Model Canon DIGITAL IXUS II	0.004	2.037	0.998	1.000

Model Canon EOS 10D	3.513	2.116	0.097	1.000
Model Canon EOS 1100D	1.143	0.424	0.007	0.350
Model Canon EOS 300D DIGITAL	-1.016	0.874	0.246	1.000
Model Canon EOS 350D DIGITAL	1.970	0.735	0.007	0.366
Model Canon EOS 400D DIGITAL	-0.089	0.587	0.879	1.000
Model Canon EOS 40D	2.235	1.456	0.125	1.000
Model Canon EOS 650D	-0.678	1.201	0.572	1.000
Model Canon EOS 7D	0.372	0.885	0.675	1.000
Model Canon EOS DIGITAL REBEL XTi	2.161	0.772	0.005	0.255
Model Canon PowerShot A75	-0.876	2.037	0.667	1.000
Model Canon PowerShot A95	-0.462	0.772	0.549	1.000
Model Canon PowerShot S1 IS	2.357	2.037	0.248	1.000
Model Canon PowerShot S2 IS	4.227	2.037	0.038	1.000
Model Canon PowerShot S3 IS	4.979	2.501	0.047	1.000
Model Canon PowerShot S40	6.149	2.620	0.019	0.932
Model Canon PowerShot S410	6.905	2.866	0.016	0.789
Model Canon PowerShot S45	1.593	1.456	0.274	1.000
ModelC anon PowerShot SX40 HS	0.283	0.949	0.765	1.000
Model DSC-HX400V	0.509	1.456	0.727	1.000
Model E-1	-0.436	1.429	0.760	1.000
Model FinePix S5000	-0.414	0.374	0.269	1.000
Model FinePix S5600	0.462	0.259	0.074	1.000
Model FinePix4700 ZOOM	0.297	1.456	0.838	1.000
Model FinePixS2Pro	-0.023	0.427	0.957	1.000
Model KODAK DX6490	1.126	0.734	0.125	1.000
Model Nikon D100	0.405	1.201	0.736	1.000
Model Nikon D300S	2.324	0.689	0.001	0.038*
Model Nikon D3100	2.367	0.450	0.000	<0.001*
Model Nikon D50	0.382	0.254	0.133	1.000
Model Nikon D70	0.864	0.298	0.004	0.188
Model Nikon D70s	-0.322	0.340	0.344	1.000
Model Pentax *ist DL	1.030	1.015	0.310	1.000
Model QV-R41	0.541	2.116	0.798	1.000

Tusk diameter: body length	Model Olympus C-740 UZ (Intercept)	0.259	0.029	2.000E-16	1.54E-16*
	Megapixels	0.004	0.005	0.484	1.000
	Aperture	-0.008	0.004	0.053	1.000
	Shutter speed	-6.414	8.005	0.423	1.000
	ISO	-5.438E-5	3.315E-5	0.102	1.000
	White balance cloudy	0.022	0.022	0.319	1.000
	White balance daylight	-0.012	0.028	0.673	1.000
	White balance manual	-0.029	0.039	0.463	1.000
	White balance shade	0.021	0.026	0.426	1.000
	White balance sunny	-0.038	0.056	0.503	1.000
	Focal length	-1.503E-4	8.236E-5	0.069	1.000
	Quality low	-0.046	0.040	0.259	1.000
	Quality normal	-0.006	0.022	0.776	1.000
	Quality super fine	0.133	0.067	0.049	1.000
	Model Olympus C-750 UZ	-0.021	0.012	0.072	1.000
	Model Canon IXUS 330	-0.047	0.035	0.172	1.000
	Model Canon IXUS 55	0.010	0.047	0.834	1.000
	Model Canon EOS 1100D	0.061	0.068	0.364	1.000
	Model Canon EOS 300D	-0.030	0.052	0.566	1.000
	Model Canon EOS 350D	0.060	0.069	0.386	1.000
	Model Canon EOS 400D	0.035	0.066	0.597	1.000
	Model Canon EOS 40D	0.129	0.081	0.110	1.000
	Model Canon EOS 650D	-0.011	0.106	0.920	1.000
	Model Canon EOS 7D	-0.008	0.112	0.945	1.000
	Model Canon PowerShot A95	-0.092	0.045	0.040	1.000
	Model Canon PowerShot S1 IS	0.212	0.090	0.019	0.827
	Model Canon PowerShot S3 IS	-0.161	0.066	0.015	0.657
	Model Canon PowerShot S40	-0.207	0.088	0.020	0.840
	Model Canon PowerShot S410	NA	NA	NA	NA
	Model Canon PowerShot SX40 HS	-0.071	0.107	0.510	1.000
	Model Olympus E-1	0.004	0.091	0.963	1.000
	Model Fujifilm FinePix S5000	-0.021	0.031	0.500	1.000
	Model Fujifilm FinePix S5600	0.015	0.023	0.527	1.000

	Model Fujifilm FinePix 4700	0.187	0.091	0.040	1.000
	Model Fujifilm FinePix S2 Pro	-0.013	0.060	0.833	1.000
	Model Nikon D300S	-0.029	0.079	0.718	1.000
	Model Nikon D3100	0.069	0.048	0.150	1.000
	Model Nikon D50	0.025	0.026	0.335	1.000
	Model Nikon D70	-0.013	0.027	0.630	1.000
	Model Nikon D70S	-0.005	0.042	0.897	1.000
	Aperture: shutter speed	-1.398	1.259	0.268	1.000
	Aperture: ISO	3.378E-5	2.193E-6	0.124	1.000
	Shutter speed: ISO	0.004	0.010	0.664	1.000
	Aperture: shutter speed: ISO	0.001	0.001	0.502	1.000
Tusk diameter: body height	Model Olympus C-740 UZ (Intercept)	0.036	0.003	2.000E-16	2.110E-37*
	Megapixels	4.568E-4	4.857E-4	0.347	1.000
	Aperture	0.001	3.682E-4	0.173	1.000
	Shutter speed	0.492	0.720	0.495	1.000
	ISO	-4.091E-6	2.980E-6	0.170	1.000
	White balance cloudy	0.003	0.002	0.181	1.000
	White balance daylight	0.001	0.003	0.636	1.000
	White balance manual	0.002	0.004	0.547	1.000
	White balance shade	0.003	0.002	0.280	1.000
	White balance sunny	-0.005	0.005	0.370	1.000
	Focal length	-5.782E-6	7.404E-6	0.435	1.000
	Quality low	-4.258E-4	0.004	0.907	1.000
	Quality normal	-0.001	0.002	0.670	1.000
	Quality super fine	0.007	0.006	0.242	1.000
	Model Olympus C-750 UZ	-0.003	0.001	0.011	0.459
	Model Canon IXUS 330	-0.001	0.003	0.806	1.000
	Model Canon IXUS 55	0.001	0.004	0.885	1.000
	Model Canon EOS 1100D	-0.004	0.006	0.521	1.000
	Model Canon EOS 300D	-0.004	0.005	0.390	1.000
	Model Canon EOS 350D	-0.002	0.006	0.737	1.000
	Model Canon EOS 400D	5.240E-5	0.006	0.993	1.000
	Model Canon EOS 40D	0.003	0.007	0.690	1.000

	Model Canon EOS 650D	-0.001	0.010	0.922	1.000
	Model Canon EOS 7D	-0.003	0.010	0.794	1.000
	Model Canon PowerShot A95	-0.014	0.004	0.001	0.034 *
	Model Canon PowerShot S1 IS	0.013	0.008	0.110	1.000
	Model Canon PowerShot S3 IS	-0.011	0.006	0.077	1.000
	Model Canon PowerShot S40	-0.009	0.008	0.271	1.000
	Model Canon PowerShot S410	NA	NA	NA	NA
	Model Canon PowerShot SX40 HS	-0.005	0.010	0.598	1.000
	Model Olympus E-1	-0.004	0.008	0.647	1.000
	Model Fujifilm FinePix S5000	0.004	0.003	0.140	1.000
	Model Fujifilm FinePix S5600	-9.794E-5	0.002	0.963	1.000
	Model Fujifilm FinePix 4700	0.020	0.008	0.017	0.726
	Model Fujifilm FinePix S2 Pro	-0.006	0.005	0.256	1.000
	Model Nikon D300S	-0.011	0.007	0.114	1.000
	Model Nikon D3100	-4.675E-4	0.004	0.913	1.000
	Model Nikon D50	-0.002	0.002	0.300	1.000
	Model Nikon D70	-0.009	0.002	3.180E-4	0.014*
	Model Nikon D70S	-0.001	0.004	0.805	1.000
	Aperture: shutter speed	-0.416	0.113	2.620E-4	0.011*
	Aperture: ISO	1.488E-7	1.971E-7	0.451	1.000
	Shutter speed: ISO	3.028E-4	0.001	0.735	1.000
	Aperture: shutter speed: ISO	1.097E-4	7.051E-5	0.120	1.000
Tusk diameter: foot diameter	Model Olympus C-740 UZ (Intercept)	0.220	0.016	2.000E-16	0.006*
	Megapixels	1.192E-4	0.003	0.969	1.000
	Aperture	0.004	0.002	0.060	1.000
	Shutter speed	1.372	4.442	0.757	1.000
	ISO	-3.134E-5	1.838E-5	0.089	1.000
	White balance cloudy	0.012	0.012	0.335	1.000
	White balance daylight	0.004	0.015	0.773	1.000
	White balance manual	0.001	0.022	0.971	1.000
	White balance shade	0.010	0.014	0.501	1.000
	White balance sunny	-0.045	0.031	0.144	1.000

Focal length	-8.205E-5	4.529E-5	0.071	1.000
Quality low	-0.003	0.023	0.879	1.000
Quality normal	-0.001	0.013	0.921	1.000
Quality super fine	0.045	0.037	0.223	1.000
Model Olympus C-750 UZ	-0.012	0.006	0.063	1.000
Model Canon IXUS 330	-0.013	0.022	0.545	1.000
Model Canon IXUS 55	0.015	0.025	0.553	1.000
Model Canon EOS 1100D	-0.004	0.039	0.924	1.000
Model Canon EOS 300D	-0.022	0.035	0.538	1.000
Model Canon EOS 350D	-0.006	0.038	0.878	1.000
Model Canon EOS 400D	0.056	0.056	0.323	1.000
Model Canon EOS 40D	0.056	0.045	0.215	1.000
Model Canon EOS 650D	0.035	0.060	0.564	1.000
Model Canon EOS 7D	0.021	0.062	0.740	1.000
Model Canon PowerShot A95	-0.070	0.030	0.018	0.782
Model Canon PowerShot S1 IS	0.028	0.050	0.575	1.000
Model Canon PowerShot S3 IS	-0.062	0.036	0.085	1.000
Model Canon PowerShot S40	-0.050	0.060	0.405	1.000
Model Canon PowerShot S410	NA	NA	NA	NA
Model Sony DSC-HX 400V	0.035	0.077	0.646	1.000
Model Olympus E-1	0.016	0.036	0.656	1.000
Model Fujifilm FinePix S5000	0.013	0.017	0.429	1.000
Model Fujifilm FinePix S5600	0.003	0.013	0.820	1.000
Model Fujifilm FinePix 4700	0.078	0.050	0.119	1.000
Model Fujifilm FinePix S2 Pro	-0.008	0.035	0.829	1.000
Model Nikon D300S	-0.029	0.045	0.513	1.000
Model Nikon D3100	0.014	0.027	0.597	1.000
Model Nikon D50	0.004	0.014	0.804	1.000
Model Nikon D70	-0.049	0.016	0.002	0.097
Model Nikon D70S	0.009	0.023	0.675	1.000
Aperture: shutter speed	-2.286	0.685	0.001	0.039*
Aperture: ISO	1.054E-6	1.211E-6	0.384	1.000
Shutter speed: ISO	0.005	0.005	0.337	1.000

Aperture: shutter speed: ISO	4.008E-4	4.287E-4	0.350	1.000
