# Supplementary material for: Mining morphometrics and age from past survey photographs
Source: Front Zool. 2019 May 13;16:14. doi: 10.1186/s12983-019-0309-x (PMC6513526; doi:10.1186/s12983-019-0309-x)
Supplement: Supplementary file 5 — Table S5. Output of generalized linear models for each of 7 explanatory variables, represented as ratios. * represents significant Bonferroni adjusted p values. Positive estimates indicate a positive relationship between coefficient and errors (ratio - mean difference derived from Bland-Altman test). (PDF 18 kb) [file 12983_2019_309_MOESM5_ESM.pdf]

Supplementary Table 5.

Response variable	Explanatory variable	Value	Standard error	DF	*p* value
	(Intercept)	-0.191	4.17	46	0.964
Head height					
	Body height	-0.007	0.007	22	0.352
	Body length	0.026	0.018	22	0.174
	Foot diameter	1.01	0.091	22	<0.001*
Head girth	(Intercept)	-1.63	5.61	178	0.772
	Body height	0.003	0.0101	178	0.763
	Body length	-0.054	0.033	178	0.106
	Foot diameter	0.797	0.161	178	<0.001*
