# Supplementary material for: Mining morphometrics and age from past survey photographs
Source: Front Zool. 2019 May 13;16:14. doi: 10.1186/s12983-019-0309-x (PMC6513526; doi:10.1186/s12983-019-0309-x)
Supplement: Supplementary file 6 — Collaring procedures. (DOCX 25 kb) [file 12983_2019_309_MOESM6_ESM.docx]

# **Supplemental Materials and Methods**

**Collaring procedures**

During collaring operations all study animals were darted by a veterinarian qualified to administer the chemical agents and perform the necessary veterinary procedures. Most collaring operations took place from a helicopter flown by a pilot experienced in wildlife capture or related procedures. The study animal, whether a bull or cow, was darted in the large muscle groups at right angles to the body surface to ensure deep intramuscular injection of the hindquarters, back or shoulders. The sedative A30-80 was administered as a mixture with M99, with the dose dependent on the age, sex and body condition of the animal. Azaperone was added to reduce hypertension, and hyaluronidase to assist with drug absorption. The drugs were administered from a dart gun at a shooting range of 30–40 m from the study animal. During collaring operations and after delivery of the drug the darted animal was separated by helicopter from the associating family or bull group. Induced animals slowed their pace under the effects of the drug, eventually toppling over within an average 10 minutes. The immobilized animal was fitted with a collar, ensuring that the collar was not fitted too tightly. Steel cable passing through an iron rod was used to pull the collar between the ear and the neck without damaging ear cartilage. Morphometric measurements of the back length, shoulder height, feet circumference and diameter were taken. Blood and tail hair samples were collected from the immobilized animals for DNA and carbon isotope analyses of short-term dietary changes, respectively. Dental impressions were taken from 11 bulls using methods based on those described by Rasmussen et. al. (2005). Speedex Putty was used as a silicone-based impression material together with Universal Activator from Coltène/Whaledent AG (Coltène, Altstsätten, Switzerland). Age estimates (accurate within ± 3 years) could then be made from all molar progressions as described by (see Laws 1966, Sikes 1971, Jachmann 198, Manspeizer & Delelegn 1992, Lee et. al 2011). In cases where the immobilized animal did not spontaneously roll onto its side (lateral recumbency) or could not be assisted by the ground crew to do so, thereby remaining in an upright ‘sitting’ position (sternal recumbency), collars were fitted as quickly as possible and the full antidote administered to ensure the well-being of the elephant as the bulk of the digestive tract and the shape of the thoracic cavity can lead to respiratory distress. As elephants are obligate nasal breathers the trunk was kept in a straight position and a twig inserted into the external opening of the trunk to ensure unobstructed breathing. During collaring operations, the elephant’s ears were folded over the eyes to protect them from harsh sunlight, dust and trauma. Pulse rate (40–50 beats p/min) and respiratory function (6–8 breaths p/min in adults) were monitored. Body temperature was kept below 41°C by dowsing the ears and body with water at regular intervals. The antidote, consisting of naltrexone in combination with diprenorphine, was administered in a blood vessel in the ear. The antidote took effect 3–5 mins after being administered, whereupon the animal got to its feet and slowly re-orientated itself, usually in the direction of the other animals from which it was separated during the collaring operation.

**References**

Jachmann, H. 1988. Estimating age in African elephants: a revision of laws’ molar evaluation technique. *Africa Journal of Ecology*, **22**, 51–56.

Laws, R.M. 1966. Age criteria for the African elephant, Loxodonta africana. *East African Wildlife Journal*, **4**, 1–37.

Lee, C.L., Sayialel, S., Lindsay, K. & Moss, C. 2011. African elephant age determination from 24 teeth: validation from known animals. *African Journal of Ecology*, **50**, 9–20.

Manspeizer, I. & Delelegn, Y. 1992. Ethiopian elephant conservation development programme field manual. Ethiopian wildlife conservation organisation.

Rasmussen, H. B., Wittemyer, G. & Douglas-Hamilton, I. 2005. Estimating age of immobilized elephants from teeth impressions using dental silicon. *African Journal of Ecology*, **43**, 215–219.

Sikes, S. K. 1971. The natural history of the African elephant. London: Weidenfeld and Nicolson.
